# A new toolkit for gene tagging in *Candida albicans* containing recyclable markers

**DOI:** 10.1371/journal.pone.0219715

**Published:** 2019-07-11

**Authors:** Encarnación Dueñas-Santero, Ana Santos-Almeida, Patricia Rojo-Dominguez, Francisco del Rey, Jaime Correa-Bordes, Carlos R. Vázquez de Aldana

**Affiliations:** 1 Instituto de Biología Funcional y Genómica (IBFG), Consejo Superior de Investigaciones Científicas/Universidad de Salamanca, Salamanca, Spain; 2 Departamento de Ciencias Biomédicas, Universidad de Extremadura, Badajoz, Spain; Louisiana State University, UNITED STATES

## Abstract

Gene manipulation and epitope tagging are essential tools for understanding the molecular function of specific genes. The opportunistic human pathogen *Candida albicans* is a diploid fungus that utilizes a non-canonical genetic code. Since selection markers available in this organism are scarce, several tools based on recyclable markers have been developed for gene disruption, such as the *Clox* system. This system relies on the Cre recombinase, which recycles selection markers flanked by *loxP* sites with high efficiency, facilitating single marker or multi-marker recycling. However, PCR-based modules for epitope tagging, such the pFA-modules, mainly use limited non-recyclable auxotrophic markers. To solve this problem, we have used a Gibson assembly strategy to construct a set of new plasmids where the auxotrophic markers of the pFA vectors were swapped with five recyclable marker modules of the *Clox* system, enhancing the versatility of the pFA plasmids. This new toolkit, named pFA-*Clox*, is composed of 36 new vectors for gene disruption and epitope tagging (GFP, 3xGFP, mCherry, 3xHA, 5xmyc and TAP). These plasmids contain the dominant *NAT1* marker, as well as *URA3*, *HIS1* and *ARG4* cassettes, thereby permitting functional analysis of laboratory strains as well as clinical isolates of *C*. *albicans*. In summary, we have adapted the *Clox* system to the pFA-backbone vectors. Thus, the set of primers used for the amplification of previously published pFA modules can also be utilized in this new pFA-*Clox* system. Therefore, this new toolkit harbors the advantages of both systems, allowing accelerated gene modification strategies that could reduce time and costs in strain construction for *C*. *albicans*.

## Introduction

*Candida albicans* is an opportunistic human pathogen that lives as a commensal in the epithelium of the oral cavity, gastrointestinal or urogenital tracts [1]. Due to the particular characteristics of its biology, gene manipulation is a time-consuming process that requires the development of *Candida*-specific tools. First, *C*. *albicans* is mainly a diploid with a particular parasexual life cycle that does not seem to undergo meiosis to complete a standard sexual cycle [2,3]. For that reason, the generation of homozygous deletion mutants requires the successive deletion of both alleles. If more than one locus is going to be deleted, then the genetic markers used to select transformants need to be recycled [4–6]. The second important aspect of its biology is the use of a non-canonical genetic code in which CTG is decoded as Ser instead of Leu [7], which requires the development of codon-optimized tools for this organism (reviewed in [8,9]).

Over the last years, a variety of tools for gene manipulation have been developed for *C*. *albicans*, allowing genetic approaches to study function of the genes, including gene disruption [6,10–13], protein tagging [11,14,15] and overexpression [12,15]. In all the cases, these techniques are based on the generation of a cassette by PCR that is integrated in the genome by homologous recombination between sequences in the cassette and the genomic sequences.

The *URA* blaster method was the classical approach to disrupt genes in *C*. *albicans*. This cassette carries two direct repeats of the *hisG* sequence from *Salmonella typhimurium* flanking the *URA3* gene [4]. After transformation of a *ura3/ura3* strain, Uri^+^ transformants are selected on uracil-deficient medium. Then, spontaneous recombination between two *hisG* repeats produces the excision of the marker, leaving behind a copy of the *hisG* sequence in the genome. *Ura3*^*-*^ cells can be selected by growth on medium containing 5-fluoroorotic acid (5-FOA), since it is toxic to Uri^+^ cells. However, 5-FOA is potentially mutagenic and could introduce chromosomal rearrangements in *C*. *albicans* [16,17].

To circumvent this problem, strains with additional selectable markers were generated on the SC5314 background, such as BWP17 (Uri^-^ His^-^ Arg^-^), SN76 (Uri^-^ His^-^ Arg^-^), SN78 (Uri^-^ His^-^ Leu^-^) or SN148 (Uri^-^ His^-^ Arg^-^ Leu^-^) [18,19], and several systems with recyclable markers were also developed. The *URA* flipper cassette contains two direct repeats of the minimal *FLP* recombination target (FRT) sequence flanking the *URA3* marker. This cassette also contains the *FLP* recombinase gene under the control of the secreted aspartyl proteinase (*SAP2*) promoter, which is induced by growth in medium containing bovine serum albumin. The *FLP* recombinase promotes the homologous recombination between the *FRT* sites to recycle the *URA3* gene for another round of transformation [20]. Similar systems are the *SAT1* flipper, which contains the nourseothricin resistance marker *SAT1* and the *FLP* recombinase regulated by the *MAL2* promoter [21], and a variant that contains the *NAT1* marker, which is a codon-optimized *Streptomyces noursei NAT1* gene (nourseothricin acetyltransferase) that also confers nourseothricin resistance regulated by the *Ashbya gossypii TEF1* promoter followed by the *FLP* recombinase gene regulated by the *SAP2* promoter [22].

A *Cre-loxP* system for gene disruption and marker recycling in *C*. *albicans* was developed a few years ago [23]. Cre catalyzes the recombination between *loxP* sequences in P1 bacteriophage [24]. The specificity of sequence recognition has been used to develop different *Cre*-*loxP* recombination tools for *Saccharomyces cerevisiae* and mammalian cells [25,26]. The *C*. *albicans* system also involves the use of Cre recombinase to recycle different selection markers flanked by *loxP* sites [23]. Three different selectable markers flanked by *loxP* sequences were developed: *loxP-ARG4-loxP* (LAL), *loxP-HIS1-loxP* (LHL) and *loxP-URA3-loxP* (LUL). The initial system used a methionine-regulatable *MET3p*-*cre* cassette to induce marker loss that had to be inserted in an additional transformation step after the two target alleles have been disrupted. More recently, an enhanced *Cre-loxP* toolkit (*Clox*) was developed [13], which carries a synthetic, intron-containing *cre* gene under the control of the *MET3p* promoter and the *URA3* or *NAT1* selectable markers flanked by *loxP* sequences (*URA3-Clox* or *NAT1-Clox* cassettes). The advantage of these new cassettes is that they facilitate gene disruption using a single marker or multiple markers, with high efficiency of marker loss after induction of the Cre recombinase. The only disadvantage of this toolkit is that the sequences flanking the cassettes used for amplification are different from the commonly used sequences present in pFA plasmids developed for *Candida* [11,12,14,15], and therefore they require the design of new sets of oligonucleotides for their use.

The development of clustered regularly interspaced short palindromic repeat(s) (CRISPR) genome editing systems has changed genetic engineering in different species (reviewed in [27]). Several groups have reported the development of CRISPR-mediated editing systems for *C*. *albicans* [28–30]. These systems generate a Cas9-mediated double-stranded breaks (DSBs) at specific target locus that are repaired by the integration of a donor DNA (dDNA). These new systems are a breakthrough in *C*. *albicans* genome manipulation since a homozygous gene deletion mutant is obtained in a single transformation. Recently, an optimized system has been described that allows high-efficiency marker-less homozygous genome editing, rapid cloning-free gRNA generation and marker recycling [31]. In addition, the CRISPR system has also been adapted to regulate gene expression in *C*. *albicans* using a Cas9-inactive nuclease [32,33].

Regarding to epitope tagging with recyclable markers, the number of plasmids for epitope tagging that allows marker recycling is more limited. Plasmids containing the 13xmyc epitope or a 6-His/FLAG tandem affinity purification (TAP) tag and the *SAT1* flipper cassette have been described [34]. More recently, a collection of vectors for epitope-tagging and overexpression in *C*. *albicans* based on the Cre recombinase was also described [35]. Here we describe the development of the pFA-*Clox* toolkit, a new collection of plasmids for gene deletion and epitope tagging that use the selectable markers of the *Cre-loxP* system cloned into the pFA vector backbone. This new toolkit combines the advantages of the Clox system (rapid, efficient and flexible gene disruption and marker recycling in *C*. *albicans*)[13] with the use of the common flanking regions of the pFA vectors, thereby reducing the need of generating new sets of oligonucleotides. The different cassettes can be amplified with fewer 120-bp primer pairs (100 bp from the gene to be tagged and 20 bp of vector sequences) to tag genes with different epitopes or fluorescent proteins. This PCR strategy is compatible with the pFA-tagging systems previously published [11,12,14]. The plasmids developed here could be used to generate single or multiple deletion strains in auxotrophic laboratory strains and in prototrophic clinical isolates. They also permit simultaneous tagging of different genes with commonly used epitope tags (HA, myc, GFP, mCherry…) followed by marker excision mediated by the Cre recombinase. Consequently, strains generated using this system will have available markers for successive gene manipulations.

## Results

### The pFA-*Clox* kit

**Fig 1:**
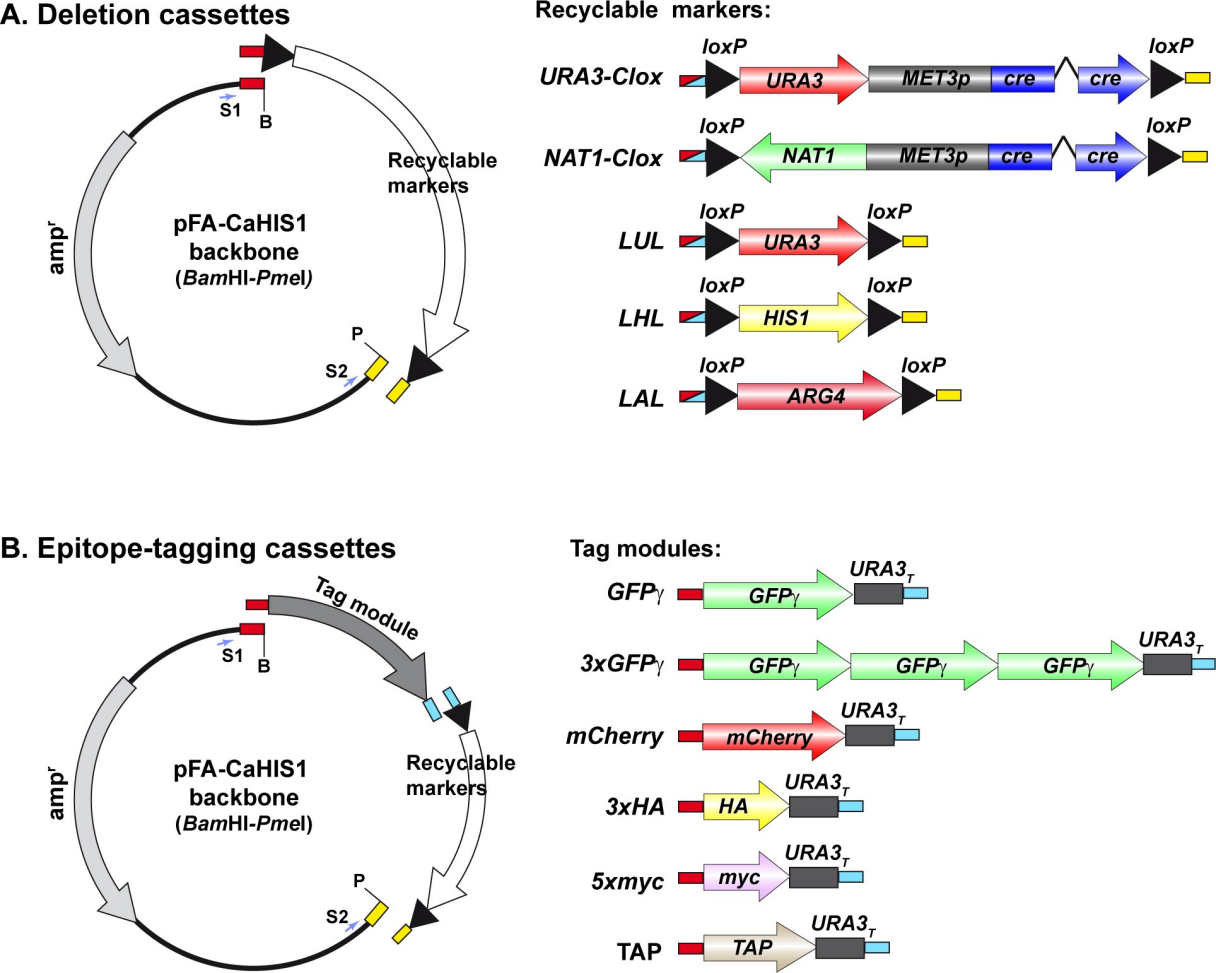
Modular strategy used for the construction of pFA plasmids with recyclable markers. (A) Construction of pFA deletion plasmids. The five recyclable markers flanked by *loxP* repeats were amplified with oligonucleotides LXL1 and LXL2, which generated overlapping ends with the pFA-CaHIS1 backbone digested with *Bam*HI and *Pme*I. The amplified fragments were assembled with the vector in 5 independent reactions using NEBuilder HiFi DNA Assembly kit. (B) Construction of epitope-tagging plasmids. The different tagging modules (GFPγ, 3xGFPγ, mCherry, 3xHA, 5xmyc or TAP-TAG) were amplified with oligonucleotides that generated overlapping ends with the pFA-CaHIS1 backbone digested with *Bam*HI (red) and with the 5´end of the recyclable marker module (light blue). The five recyclable markers were independently amplified with oligonucleotides that produced DNA fragments containing overlapping ends with the tagging modules (light blue) and the pFA-CaHIS1 backbone digested with *Pme*I (yellow). The different modules were assembled with the vector in independent reactions using NEBuilder HiFi DNA Assembly kit.

An arsenal of pFA plasmids for PCR-based gene targeting for function analysis in *C*. *albicans* has developed since 2003 by Wendland and colleagues [10–12,14]. These pFA series have been generated to delete genes, exchange promoters and tag genes with GFP, myc or HA. An advantage of the pFA series is the minimal set of primers required for the amplification of all modules, reducing the cost of strain construction. However, the use of non-recyclable selection markers diminishes the versatility of these plasmids. Recently, an improved *Clox* system has been developed that is suitable for gene disruption in *C*. *albicans* that allows both multi-marker disruption and single marker recycling strategies with high efficiency [13]. Therefore, we designed an assembly strategy to replace the non-recyclable auxotrophic markers of pFA plasmids for gene disruption and C-terminal tagging with the five recyclables modules of the *Clox* system (*LAL*, *LHL*, *LUL*, *URA3-Clox* and *NAT1-Clox*) (Fig 1A).

To generate a new pFA series with these recyclable markers for gene disruption, the pFA-CaHIS1 plasmid [11] was digested with *Bam*HI and *Pme*I to release the *HIS1* sequence and the pFA backbone was assembled with each of the five modules of the Clox system (*LAL*, *LHL*, *LUL*, *URA3-Clox* and *NAT1-Clox*) using the NEBuilder HiFI DNA Assembly Cloning Kit as described in Materials and Methods. The resulting plasmids were named pFA-LAL, pFA-LHL, pFA-LUL, pFA-URA3-Clox and pFA-NAT1-Clox (Table 1 and Figure A in S1 File). To amplify the disruption cassettes present in all these plasmids, the same pair of primers (containing S1 and S2 sequences at the ends, Table 2) can be used.

**Table 1:** The pFA-*Clox* toolkit. Plasmids generated and fragment length of the cassettes.

Plasmid	Accession number	Selectable Marker	Gene	5´ end	3´ end	Cassette Size
pFA-LAL	MK652110	*loxP-ARG4-loxP*	-	S1	S2	2180
pFA-LHL	MK652111	*loxP-HIS1-loxP*	-	S1	S2	1474
pFA-LUL	MK652112	*loxP-URA3-loxP*	-	S1	S2	1580
pFA-NAT1-Clox	MK652113	*loxP-NAT1-cre-loxP*	-	S1	S2	4576
pFA-URA3-Clox	MK652114	*loxP-URA3-cre-loxP*	-	S1	S2	4344
pFA-GFPγ-LAL	MK652105	*loxP-ARG4-loxP*	GFPγ	S1-XFP	S2	3142
pFA-GFPγ-LHL	MK652106	*loxP-HIS1-loxP*	GFPγ	S1-XFP	S2	2436
pFA-GFPγ-LUL	MK652107	*loxP-URA3-loxP*	GFPγ	S1-XFP	S2	2542
pFA-GFPγ-NAT1-Clox	MK652108	*loxP-NAT1-cre-loxP*	GFPγ	S1-XFP	S2	5538
pFA-GFPγ-URA3-Clox	MK652109	*loxP-URA3-cre-loxP*	GFPγ	S1-XFP	S2	5306
pFA-3xGFPγ-LAL	MK652100	*loxP-ARG4-loxP*	3xGFPγ	S1-XFP	S2	4576
pFA-3xGFPγ-LHL	MK652101	*loxP-HIS1-loxP*	3xGFPγ	S1-XFP	S2	3870
pFA-3xGFPγ-LUL	MK652102	*loxP-URA3-loxP*	3xGFPγ	S1-XFP	S2	3976
pFA-3xGFPγ-NAT1-Clox	MK652103	*loxP-NAT1-cre-loxP*	3xGFPγ	S1-XFP	S2	6972
pFA-3xGFPγ-URA3-Clox	MK652104	*loxP-URA3-cre-loxP*	3xGFPγ	S1-XFP	S2	6740
pFA-mCherry-LAL	MK652115	*loxP-ARG4-loxP*	yEmCherry	S1-XFP	S2	3139
pFA-mCherry-LHL	MK652116	*loxP-HIS1-loxP*	yEmCherry	S1-XFP	S2	2433
pFA-mCherry-LUL	MK652117	*loxP-URA3-loxP*	yEmCherry	S1-XFP	S2	2539
pFA-mCherry-NAT1-Clox	MK652118	*loxP-NAT1-cre-loxP*	yEmCherry	S1-XFP	S2	5535
pFA-mCherry-URA3-Clox	MK652119	*loxP-URA3-cre-loxP*	yEmCherry	S1-XFP	S2	5303
pFA-HA-LAL	MK652120	*loxP-ARG4-loxP*	3xHA	S1-HA	S2	2518
pFA-HA-LHL	MK652121	*loxP-HIS1-loxP*	3xHA	S1-HA	S2	1812
pFA-HA-LUL	MK652122	*loxP-URA3-loxP*	3xHA	S1-HA	S2	1918
pFA-HA-NAT1-Clox	MK652123	*loxP-NAT1-cre-loxP*	3xHA	S1-HA	S2	4914
pFA-HA-URA3-Clox	MK652124	*loxP-URA3-cre-loxP*	3xHA	S1-HA	S2	4656
pFA-myc-LAL	MK652125	*loxP-ARG4-loxP*	5xmyc	S1	S2	2614
pFA-myc-LHL	MK652126	*loxP-HIS1-loxP*	5xmyc	S1	S2	1908
pFA-myc-LUL	MK652127	*loxP-URA3-loxP*	5xmyc	S1	S2	2014
pFA-myc-NAT1-Clox	MK652128	*loxP-NAT1-cre-loxP*	5xmyc	S1	S2	5010
pFA-myc-URA3-Clox	MK652129	*loxP-URA3-cre-loxP*	5xmyc	S1	S2	4798
pFA-TAP-LAL	MK652130	*loxP-ARG4-loxP*	TAP	S1	S2	2986
pFA-TAP-LHL	MK652131	*loxP-HIS1-loxP*	TAP	S1	S2	2280
pFA-TAP-LUL	MK652132	*loxP-URA3-loxP*	TAP	S1	S2	2386
pFA-TAP-NAT1-Clox	MK652133	*loxP-NAT1-cre-loxP*	TAP	S1	S2	5382
pDIS-NAT1-Clox	MK652134	*loxP-NAT1-cre-loxP*	-	N5-up	N5-dw	5448
pDIS-URA3-Clox	MK652135	*loxP-URA3-cre-loxP*	-	N5-up	N5-dw	5216

**Table 2:** Primers used.

**For module construction**	
Primer	Primer sequence
LXL1	TCGTACGCTGCAGGTCGACGGGCGGCCGCTCTAGAACT
LXL2	CGATGAATTCGAGCTCGTTTTTCCTGCAGATTACCCTGTTATCC
GFP-LXL1	TCGTACGCTGCAGGTCGACGGGTGCTGGCGCAGGTGCT
GFP-LXL2	AGCGGCCGCCGGATCTATGCGTCCATCTTTACAGTCCTGTC
GFP-LXL3	GCATAGATCCGGCGGCCGCTCTAGAACT
HA1	TCGTACGCTGCAGGTCGACGGGTCGACGGATCCCCGGG
Myc1	TCGTACGCTGCAGGTCGACGGATCCCCGGGGAACAGAAGCTTATATC
TAP1	TCGTACGCTGCAGGTCGACGGATCCCCGGGTTAATTAATC
NEUT7	TACCGAATTCGAGCTCGTTTGGCGGCCGCTCTAGAACT
NEUT8	GGATCCACTAGTTCTAGAGCTTCCTGCAGATTACCCTGTTATCC
**PCR primers used to amplify the transformation cassettes**	
Primer	Primer sequence^a^
S1	(100 bp target sequence)-GAAGCTTCGTACGCTGCAGGTC
S2	(100 bp target sequence)-TCTGATATCATCGATGAATTCGAG
S1–XFP	(100 bp target sequence)-**GGT GCT GGC GCA GGT GCT** TC
S1-HA	(100 bp target sequence)-**GGT CGA CGG ATC CCC GGG TAC CCA**
N5-up	CCTTAACCCACTGAATTCTACATCGAA
N5-dw	CAAAGAGAAAGCTCGGAGGAGGCT

Primer sequences are given in the 5´ to 3´ direction

^a^ The reading frame is indicated by spacing within the sequence; the sequence encoding the (Gly-Ala)_3_ linker or HA is shown in **bold**.

Next, we adapted the Clox system for C-terminal tagging with GFP and different epitopes such HA, myc and TAP. To this end, two different DNA fragments were combined in the assembly reaction with the pFA backbone, one containing the fluorescent protein/epitope sequence and the other carrying the recyclable marker (Fig 1B). Two pairs of primers were used to amplify the epitopes and the marker modules with overlapping regions for the assembly reaction (Table 2).

Using this approach, we generated three sets of plasmids for C-terminal tagging with fluorescent proteins. All pFA-*Clox* modules of these series can be amplified with the same set of primers (containing S1–XFP and S2 sequences at the ends, Table 2). The first group contain the photostable variant of the green fluorescent protein (GFPγ) [36] (pFA-GFPγ-LAL, pFA-GFPγ-LHL, pFA-GFPγ-LUL, pFA-GFPγ-URA3-Clox and pFA-GFPγ-NAT1-Clox) (Table 1 and Figure A in S1 File). The GFPγ protein is codon-optimized for *Candida* and contains 4 mutations (F64L, S65C, V163A, I167T) that result in a GFP protein with high signal intensity and photostability. In order to tag low abundant proteins, a second set of plasmids was made harboring three in-tandem copies of GFPγ (pFA-3xGFPγ-LAL, pFA-3xGFPγ-LHL, pFA-3xGFPγ-LUL, pFA-3xGFPγ-URA3-Clox and pFA-3xGFPγ-NAT1-Clox, Table 1 and Figure A in S1 File). The 3xGFPγ in tandem fusion was amplified from plasmid pFA-3xGFPγ-URA3, previously described [37]. Finally, the third set of plasmids (pFA-mCherry-LAL, pFA-mCherry-LHL, pFA-mCherry-LUL, pFA-mCherry-URA3-Clox and pFA-mCherry-NAT1-Clox, Table 1 and Figure A in S1 File) was developed to allow double color tagging of different proteins for fluorescence microscopy. The yEmCherry fluorescent protein sequence was obtained from plasmid pFA-yEmCherry-CaURA3 [14].

Next, analogous plasmids for C-terminal epitope tagging were generated. The HA and myc epitopes are highly immunogenic peptides that can then be used for Western blot or immunoprecipitation experiments. The TAP tag was developed to allow purification of protein complexes for mass spectrometry [38]. Plasmids pFA-HA-LAL, pFA-HA-LHL, pFA-HA-LUL, pFA-HA-NAT1-Clox and pFA-HA-URA3-Clox (Table 1 and Figure A in S1 File) were constructed by assembling a PCR fragment that contained 3 copies of the HA epitope followed by the *S*. *cerevisiae URA3* terminator obtained from plasmid pFA-HA-URA3 [15] with the five recyclable markers into the pFA backbone. The integration cassettes can be amplified from these plasmids with primers containing S1-HA and S2 sequences (Table 2). For plasmids pFA-myc-LAL, pFA-myc-LHL, pFA-myc-LUL, pFA-myc-NAT1-Clox and pFA-myc-URA3-Clox (Table 1 and Figure A in S1 File), a PCR fragment containing 5 copies of the myc epitope followed by the *S*. *cerevisiae URA3* terminator obtained from plasmid pFA-myc-URA3 [15] was assembled with the selection markers into the same vector. The integration cassettes can be amplified from this collection of plasmids with primers containing S1 and S2 sequences (Table 2). Finally, plasmids pFA-TAP-LAL, pFA-TAP-LHL, pFA-TAP-LUL and pFA-TAP-NAT1-Clox (Table 1 and Figure A in S1 File) contained the TAP tag followed by the *S*. *cerevisiae URA3* terminator obtained from plasmid pFA-TAP-URA3 [15]. The integration cassettes can be amplified from the four plasmids with primers containing S1 and S2 sequences (Table 2).

Finally, we constructed plasmids pDIS-URA3-Clox and pDIS-NAT1-Clox by replacing the *NAT1* marker of plasmid pDIS3 [39] with the *URA3-Clox* or *NAT1-Clox* cassettes. These plasmids can be used to induce marker loss of any construction generated with the LAL, LHL, or LUL cassettes, allowing the generation of auxotrophic strains.

### Testing deletion plasmids

**Fig 2:**
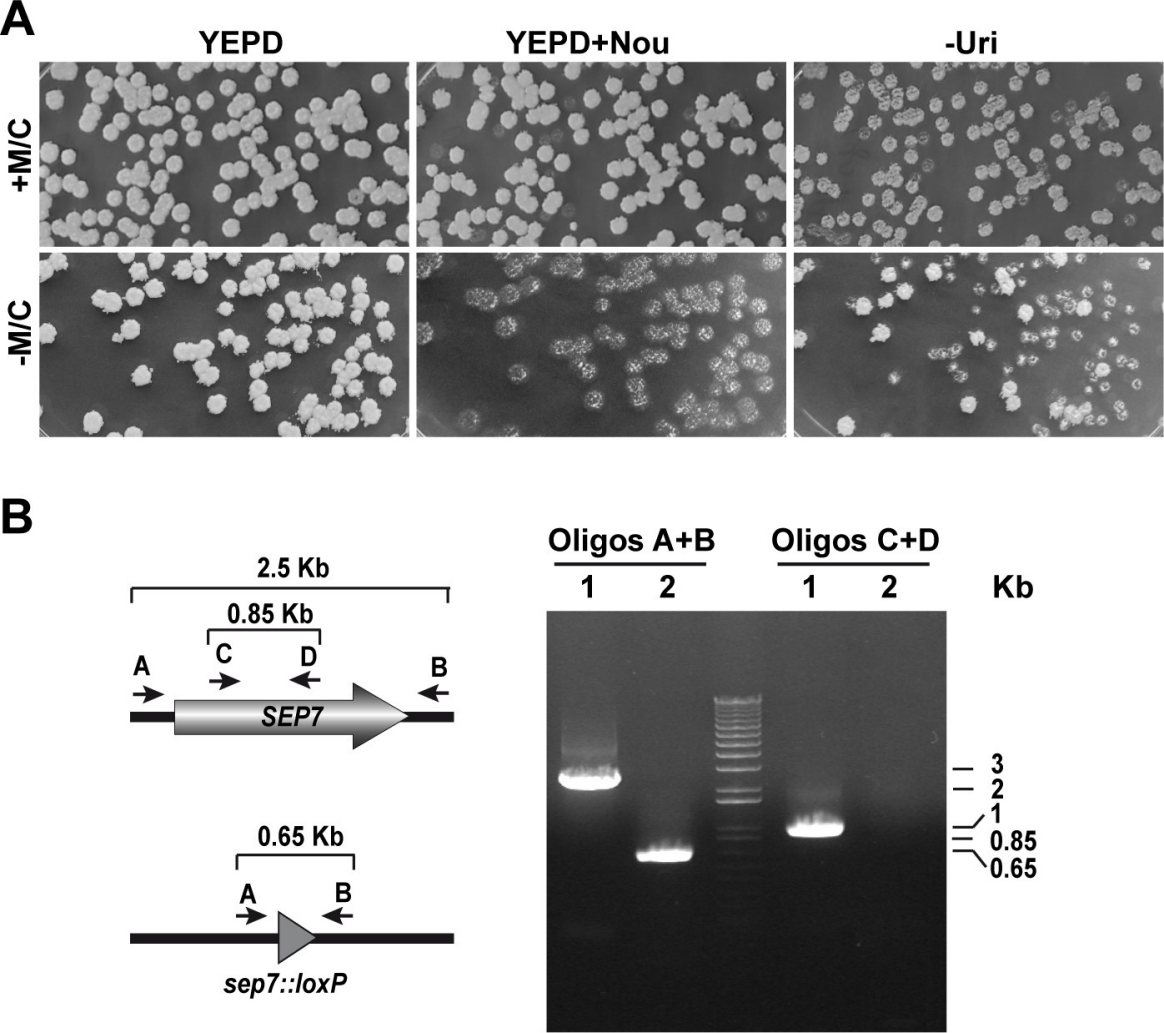
Construction of deletion mutants without selection markers. **(A)** The *sep7*::*LUL/sep7*::*NAT1-Clox* strain (OL2908) was grown overnight in YPD medium with (+M/C) or without (-M/C) methionine and cysteine to repress or induce the expression of the Cre recombinase respectively, and then plated on YPD medium. After 24 h of incubation at 28ºC, the plate was printed onto YPD+ nourseothricin or SC -Uri plates and incubated another 24 h. **(B)** PCR analysis of the different strains with specific *SEP7* oligonucleotides. Genomic DNA was isolated from the parental strain BWP17 (1) and the *sep7*::*loxP/sep7*::*loxP* (2, OL2909) mutant and analyzed with two different oligonucleotide pairs, one annealing at the 5´ and 3´ flanking regions (A and B) and another internal to the coding region (C and D) to confirm correct integration and excision of the deletion cassettes. The position of the primers in the *SEP7* locus and the *sep7*::*loxP* allele is depicted to the left.

To test the pFA-*Clox* plasmids for gene disruption, we used them to delete both copies of *SEP7*, which encodes a non-essential septin subunit that plays a regulatory role for septin function during hyphal growth [40,41]. To delete the first *SEP7* allele, BWP17 cells were transformed with a *sep7*::*LUL* cassette, amplified using long oligonucleotides carrying S1 and S2 sequences at the 3´ end and 100 nucleotides upstream or downstream of the *SEP7* gene, and transformants were selected on SC lacking uridine. To eliminate the second *SEP7* allele, the resultant Uri^+^ strain (*SEP7/sep7*Δ::*LUL*) was then transformed with the *NAT1-Clox* cassette. Nourseothricin resistant (Nou^R^) transformants were selected on YPD supplemented with nourseothricin, methionine and cysteine to repress *MET3p-cre* expression. To induce the loss of the *LUL* and *NAT1-Clox* cassettes, Uri^+^ Nou^R^ isolates (*sep7*Δ::*LUL/sep7*Δ::*NAT1-Clox*) were grown overnight in YPD without supplements and plated onto YPD to obtain single colonies. To identify Uri^-^ Nou^S^ cells, the colonies were replica plated onto YPD + nourseothricin and SC -Uri plates (Fig 2A). Interestingly, the *NAT1-Clox* cassette was resolved in all colonies tested whereas the *LUL* cassette was recycled in around 66% of the colonies. Growth of the Uri^+^ Nou^R^ isolates overnight in YPD with methionine and cysteine showed that marker resolution due to leaky cre recombinase expression is lower than 5% (Fig 2A). Similar high efficiency was obtained when the *URA3-Clox* cassette was used to construct a homozygous *sep7*Δ*/sep7*Δ null mutant from a *sep7*::*LAL/SEP7* strain (not shown). Functionality of the pDIS-URA3-Clox and pDIS-NAT1-Clox plasmids to induce marker loss after integration of the cassettes at the *NEUT5L* locus was also tested, and the frequency of marker loss was similar to that obtained with pFA-URA3-Clox or pFA-NAT1-Clox plasmids. The loss of *URA3* and *NAT1* genes in Uri^-^ Nou^S^ isolates was confirmed by diagnostic PCR using two different pairs of oligonucleotides, one annealing at the 5´ and 3´ flanking regions and another internal to the *SEP7* coding region (Fig 2B).

### Tagging multiple genes with fluorescent proteins and recyclable markers

**Fig 3:**
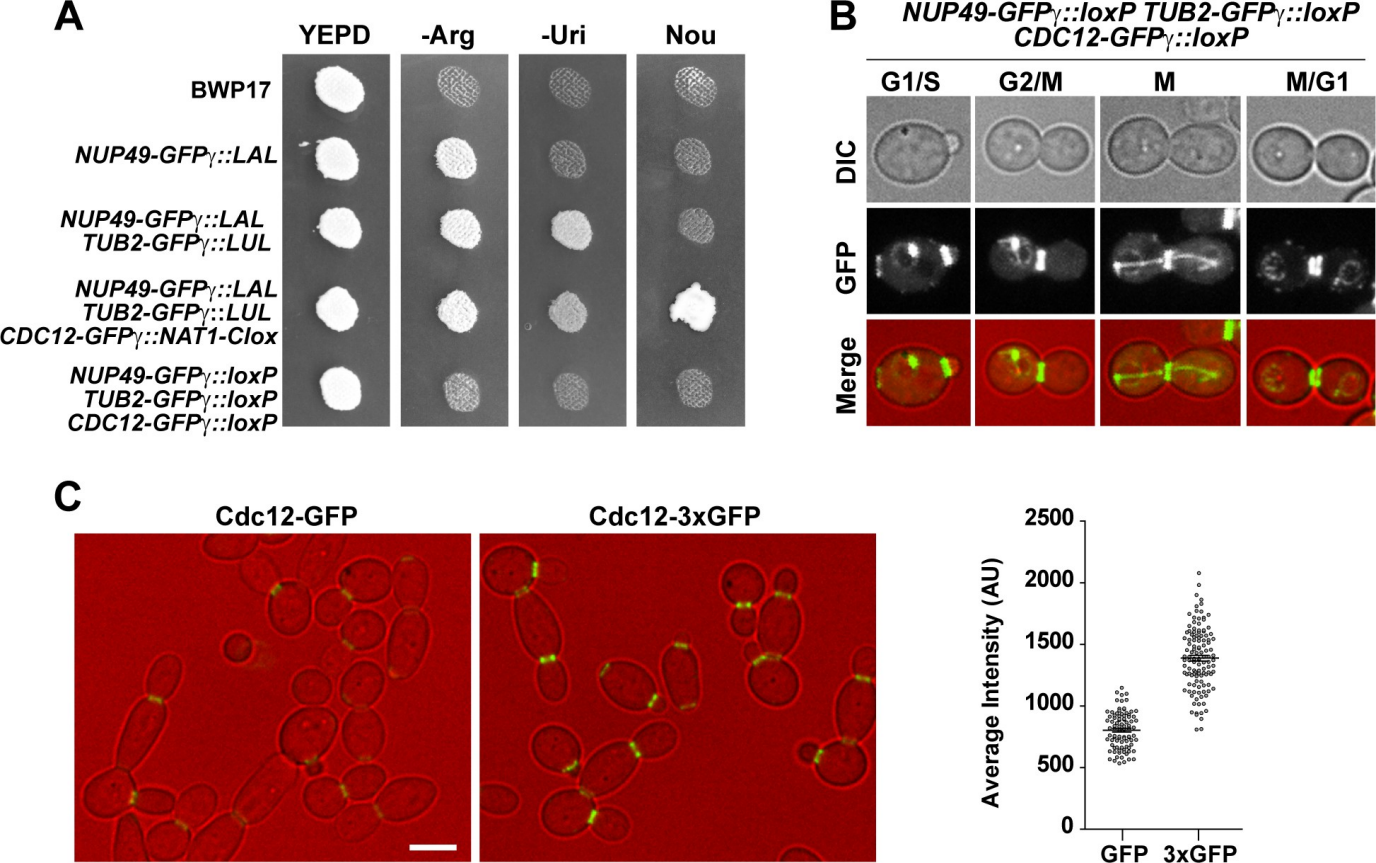
Generation of auxotroph strains containing three genes tagged with GFPγ. **(A)** Growth in selective media. Single colonies of strains BWP-17, *NUP49-GFPγ*::*LAL/NUP49* (OL2845), *NUP49-GFPγ*::*LAL/NUP49 TUB2-GFPγ*::*LUL/TUB2* (OL2847), *NUP49-GFPγ*::*LAL/NUP49 TUB2-GFPγ*::*LUL/TUB2 CDC12-GFPγ*::*NAT1-Clox/CDC12* (OL2867) and the resolved strain *NUP49-GFPγ*::*loxP/NUP49 TUB2-GFPγ*::*loxP/TUB2 CDC12-GFPγ*::*loxP/CDC12* (OL2866) were grown on YPD plates and replica-printed to -Arg, -Uri and YPD + nourseothricin media. Plates were incubated 2 days at 28ºC. **(B)** Images of cells carrying Nup49-GFPγ, Tub2-GFPγ and Cdc12-GFPγ during yeast growth. Exponential growing cultures of the *NUP49-GFPγ*::*loxP/NUP49 TUB2-GFPγ*::*loxP/TUB2 CDC12-GFPγ*::*loxP/CDC12* strain (OL2866) were imaged. The images are the maximum projection of 5 planes and show the differential interference contrast image (DIC), the GFP channel and the merged image (DIC in red, GFP in green). Scale bar, 2 μm. **(C)** Comparison of GFPγ and 3xGFPγ intensity. Images of *CDC12-GFPγ*::*LAL/CDC12 or CDC12-3xGFPγ*::*LAL/CDC12* cells during yeast growth. The images are the maximum projection of 3 planes and show the differential interference contrast image (DIC, in red) and the GFP signal (green). Scale bar, 2 μm. The graph represents the average intensity of the signal ± s.e.m. in both strains. At least 50 single rings were measured in each strain.

To validate the pFA-*Clox* plasmids for fluorescence microscopy, we sequentially tagged the nuclear membrane protein Nup49 [42] and the β-tubulin Tub2 [43] with the GFPγ-LAL and GFPγ-LUL cassettes to generate *NUP49-GFPγ TUB2-GFPγ* cells in a BWP17 background. Then, Arg^+^ Uri^+^ cells were transformed with a GFPγ-NAT1-Clox cassette to C-terminal tag the Cdc12 septin subunit [41]. Transformants were selected on YPD plus nourseothricin supplemented with methionine and cysteine to repress the *MET3p-cre* expression. As expected, cells sequentially transformed with these cassettes were Arg^+^, Uri^+^ and Nou^R^ (*NUP49-GFPγ*::*LAL TUB2-GFPγ*::*LUL CDC12-GFPγ*::*NAT1-Clox*). To induce the loss of the three selection markers, Arg^+^ Uri^+^ Nou^R^ cells were grown overnight at 30ºC in YPD lacking methionine and cysteine. Then, cells were plate on YPD and replica-plated onto selective media. Cells auxotrophic for arginine and uridine and sensitive to nourseothricin were selected (Fig 3A). As expected, the nuclear membrane, microtubules and the septin ring at the bud neck were fluorescence-labeled in the resulting strain (*NUP49-GFPγ*::*loxP TUB2-GFPγ*::*loxP CDC12-GFPγ*::*loxP*) (Fig 3B).

To check the pFA-3xGFPγ plasmids, we tagged the *CDC12* septin with one or three in-tandem copies of GFPγ to compare the intensity of the fluorescence signal in asynchronous cell cultures. To this end, BWP17 cells were transformed with the corresponding cassettes to generate the *CDC12-3xGFPγ*::*LAL/CDC12* and *CDC12-GFPγ*::*LAL/CDC12* strains. Then, the GFP signal in single septin rings of log-phase cells grown in YPD was quantified (n>50). As expected, fluorescence intensity was higher in *CDC12-3xGFPγ*::*LAL/CDC12* cells (around 2-fold higher) than in cells expressing Cdc12 tagged with a single GFPγ (*CDC12-GFPγ*::*LAL/CDC12*) (Fig 3C). Thus, the set of pFA-3xGFPγ-Clox plasmids enhance the signal of the tagged proteins and might be used to study low-expressed proteins in *C*. *albicans*.

### Dual-color fluorescence labeling with the recyclable marker plasmids

**Fig 4:**
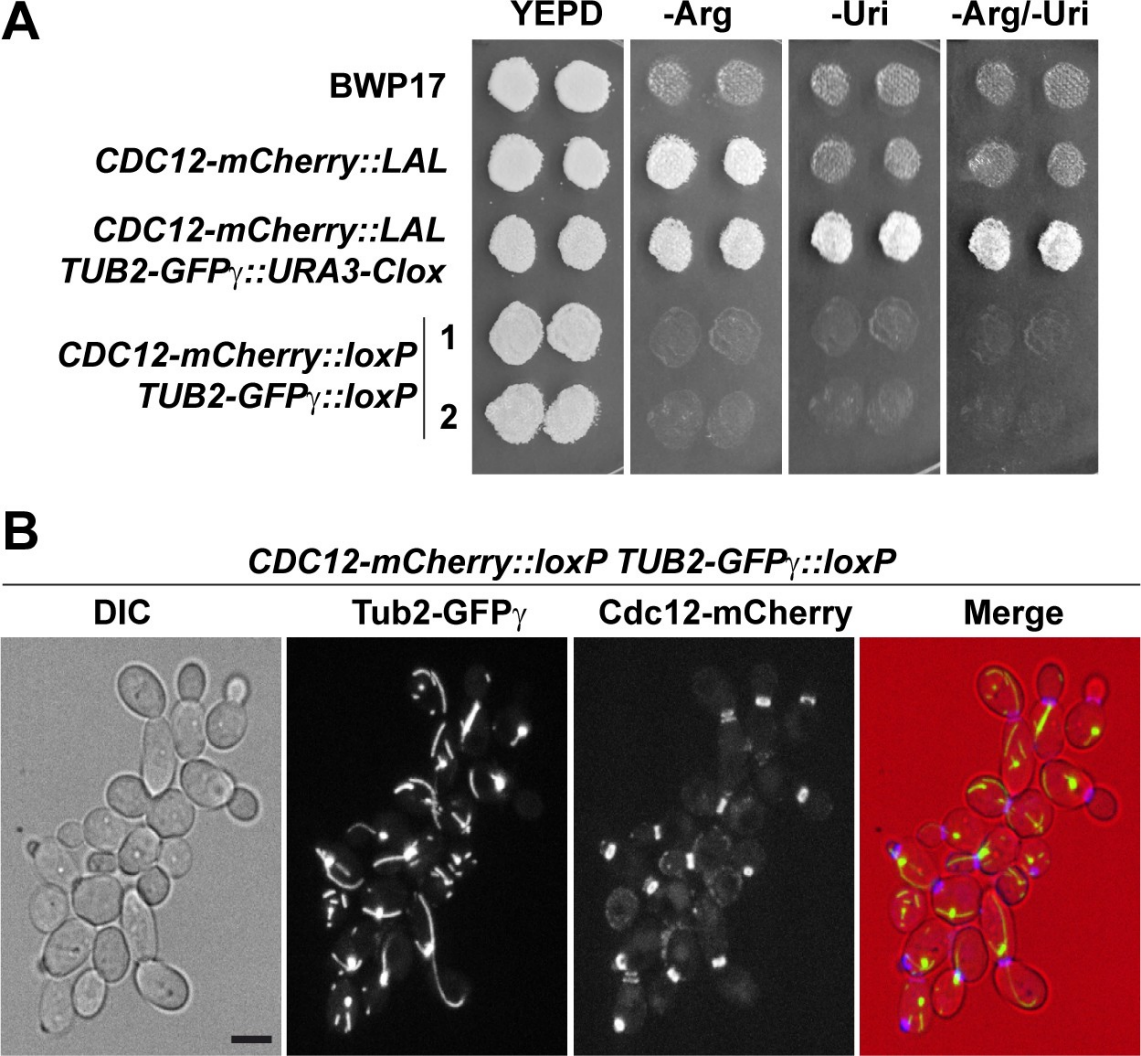
Double fluorescent tagging with GFPγ and mCherry. **(A)** Growth in selective media. Single colonies of strains BWP-17, *CDC12-mCherry*::*LAL*, *CDC12-mCherry*::*LAL TUB2-GFPγ*::*URA3-Clox* and two independent clones of the resolved strain *CDC12-mCherry*::*loxP TUB2-GFPγ*::*loxP* (1 and 2) were grown on YPD plates and replica-printed to SC -Arg, SC -Uri or SC -Arg/-Uri media. **(B)** Localization of Cdc12-mCherry and Tub2-GFP*γ* during yeast growth. Exponential growing cultures of the *CDC12-mCherry*::*loxP TUB2-GFPγ*::*loxP* strain were imaged. The images are the maximum projection of 5 planes and show the differential interference contrast image (DIC), the Tub2-GFPγ and the Cdc12-mCherry channels, and the merged image (DIC in red, Tub2-GFPγ in green and Cdc12-mCherry in blue). Scale bar, 2 μm.

We also tested the utility of the plasmids for tagging proteins with two different fluorescent markers. To this end, the wild-type strain BWP-17 was transformed with a PCR amplified *mCherry-LAL* cassette to tag the C-terminus of *CDC12*. Transformants were selected in medium lacking arginine and single colonies were isolated. The *CDC12-mCherry*::*LAL* strain was subsequently transformed with a *GFPγ-URA3-Clox* cassette to be integrated at the C-terminus of the *TUB2* gene. Uri^+^ transformants (*CDC12-mCherry*::*LAL TUB2*-*GFPγ*::*URA3-Clox*) were selected on SC lacking uridine and containing methionine and cysteine. To recycle both markers, *cre* expression was induced by growing Arg^+^ Uri^+^ cells overnight in YPD. Cells were then plated on YPD and colonies that contained only the *loxP* sequences were selected by replica-plating to selective media (Fig 4A). As expected, we were able to monitor microtubules and septin rings at the same time in these Arg^-^ Uri^-^ cells (*CDC12-mCherry*::*loxP TUB2-GFPγ*::*loxP*) (Fig 4B). Therefore, this result validates the usefulness of pFA-GFPγ-Clox and pFA-mCherry-Clox plasmids to generate strains for double labelling experiments.

### Tagging multiple genes with HA

**Fig 5:**
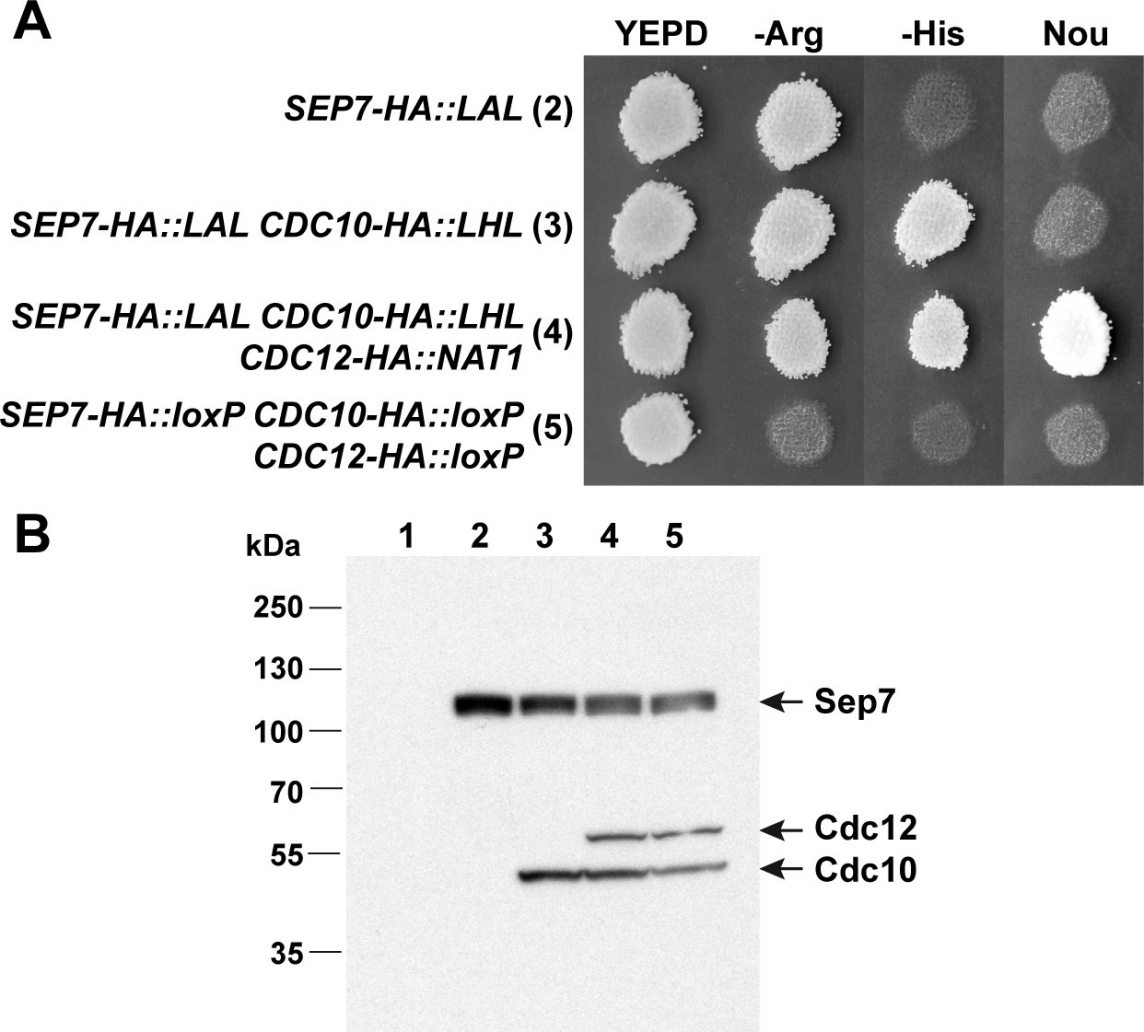
Multiple HA tagging. **(A)** Growth in selective media. Single colonies of strains *SEP7-HA*::*LAL/SEP7* (2), *SEP7-HA*::*LAL/SEP7 CDC10-HA*::*LHL/CDC10* (3), *SEP7-HA*::*LAL/SEP7 CDC10-HA*::*LHL/CDC10 CDC12-HA*::*NAT1-Clox/CDC12* (4) and *SEP7-HA*::*loxP/SEP7 CDC10-HA*::*loxP/CDC10 CDC12-HA*::*lox/CDC12* (5) were grown on YPD plates and replica-printed to SC -Arg, SC -His or YPD+ nourseothricin media. **(B)** Western blot analysis of the same strains using anti-HA antibody. The BWP17 parental strain (1) was included as an untagged control. Protein extracts were separated on 4–12% Bis-Tris gels, blotted to PDVF membranes and incubated with anti-HA antibody.

The collection of plasmids carrying HA epitopes and different recyclable markers was also tested. To this end, three of the five septin genes were tagged with the HA epitope at the C-terminus. Integration cassettes were generated using specific oligonucleotides containing 100 bp of the target sequence and either S1-HA or S2 extensions (Table 2), using different plasmids as templates. First, the wild-type strain BWP-17 was transformed with a PCR-amplified *HA-LAL* cassette to tag the C-terminus of *SEP7* to generate the *SEP7-HA*::*LAL/SEP7* strain. Then, positive colonies were transformed with a *CDC10-HA*::*LHL* cassette to construct the *SEP7-HA*::*LAL/SEP7 CDC10-HA*::*LHL/CDC10* strain, and finally, the *CDC12* gene was tagged using a *CDC12-HA*::*NAT1-Clox*. After marker resolution, colonies carrying *loxP* sequences were selected by plating colonies in YPD and replica-printing to selective media (Fig 5A). Western blot analysis of the different strains showed that the three septins were correctly tagged (Fig 5B). Similar results were obtained using the plasmids containing the myc epitope.

## Discussion

Gene deletion in *C*. *albicans* is a relatively time consuming process due to the particular characteristics of its biology. First, this fungus is diploid, being necessary to delete both chromosomal alleles to generate a homozygous null mutant. Second, it uses a non-canonical genetic code in which the CTG codon is read as Ser instead of Leu [7]. Therefore, specific codon-optimized tools have been developed for generating deletion mutants or epitope-tagged strains [8,9].

A large collection of PCR-based modules for efficient gene disruption has been developed in *C*. *albicans*. Most of them are based on the pFA-vector backbone used in the *Saccharomyces cerevisiae* EUROFAN deletion project [44]. These vectors are used to generate cassettes with flanking regions homologous to the gene of interest that target the module to the desired chromosomal locus [6,11]. In addition, the existing pFA-modules allow promoter-exchange experiments and tagging of genes with epitopes or fluorescent proteins at 5’ or 3’ end to monitor *in vivo* localization [12,14,15,45,46]. Due to the modular design of the plasmids, an advantage of this pFA system is that a minimal set of primers is required for the amplification of the cassettes, reducing the cost of strain construction.

Molecular techniques for gene manipulation in *C*. *albicans* face the problem of the limited range of auxotrophic markers available in laboratory strains (*URA3*, *HIS1*, *ARG4*, *LEU2*) [4,6,18,47] and the reduced number of dominant antibiotic markers for prototrophic clinical strains (*MPA*^*R*^, *SAT1*/*NAT1*, *HygB*)[21,22,48,49]. To circumvent this problem, strategies that allow the recycling of selectable markers have been used. Currently, the original *URA3* blaster method [4] has been replaced by recombinase-mediated systems, such as the FLP recombinase gene disruption system or the *Clox* toolkit [13,20,21,23]. FLP expression is regulated by inducible promoters to mediate site-specific recombination between the FRT sites that flank the *URA3* or *SAT1* selection markers. The *Clox* toolkit also contains several selectable markers that can be recycled after induction of the Cre recombinase, increasing the yields of desired mutants and significantly decreasing the time required to generate these mutants.

In this work, we have replaced the non-recyclable markers of the pFA plasmids with the selectable markers of the *Clox* system, which facilitate marker recycling during strain construction. We report 36 new plasmids for gene disruption and protein tagging with GFP, mCherry, 3xHA, 5xmyc and TAP tags that facilitate PCR-based genetic modifications of both laboratory strains and clinical isolates of *C*. *albicans*. The usefulness of these plasmids is that it combines the advantages of both pFA and *Clox* systems, since amplification of the modules harboring recyclable markers can be performed with a minimal set of gene-specific primers. Since we have cloned the five cassettes of the *Clox* system into the pFA backbone, the primer sets already used with published pFA series are valid for these new pFA-*Clox* plasmids. Recently, a similar vector system for C-terminal and N-terminal epitope-tagging and for inducible and constitutive promoter replacement has been described [35]. However, they were constructed in the pUC19 backbone, and therefore contain different flanking sequences for cassette amplification that are not compatible with the pFA series. In addition, the system cannot be used in prototrophic clinical strains since plasmids do not include a dominant antibiotic marker such *SAT1*.

We have validated the deletion cassettes (Fig 2) and found that marker resolution occurs with a similar efficiency to that described previously [13]. Importantly, the retrospective genotyping approach used by Shahana and colleagues is also possible with the new plasmids, and PCR diagnosis to confirm the desired homozygous null mutants can be performed after resolution of the two selection markers. Generally, it is enough to analyze several colonies from a few independent transformants to obtain the homozygous mutant.

Plasmids containing fluorescent proteins and recyclable markers have also been constructed. Three sets were generated harboring the photostable variant of the green fluorescent protein known as GFPγ [36], three in-tandem copies of GFPγ [37] or the yEmCherry fluorescent protein [14]. Given all of them are flanked by the same S1-XFP and S2 sequences, the different cassettes can be amplified with the same pair of oligonucleotides. Using these modules, we have generated strains expressing one or several tagged proteins with an efficient resolution of the selectable markers after induction of the Cre recombinase (Figs 3 and 4). For single gene tagging, the fastest approach is to use the plasmids containing either *URA3-Clox* or *NAT1-Clox* markers to induce marker resolution after transformation. To tag several genes, plasmids containing the *LAL*, *LHL* or *LUL* should be used for the initial rounds of transformation, leaving the *URA3-Clox* or *NAT1-Clox* markers for the final transformation. In this way, all the markers present in a strain can be resolved with a single step, generating the auxotroph strain carrying the tagged proteins.

For epitope tagging, we have constructed plasmids that allow the generation of cassettes to tag proteins with 3xHA, 5xmyc and TAP. Similar to the plasmids containing fluorescent proteins, they can be used individually or in different combinations to construct multiple tagged strains without selection markers in the genome.

Finally, two additional plasmids have been generated, pDIS-URA3-Clox and pDIS-NAT1-Clox. These plasmids target the cassette containing the Cre recombinase to the large intergenic region of *NEUT5L* locus, which facilitates the integration. It has been shown that integration at *NEUT5L* has no negative effect on growth and allows expression of ectopic genes, making it ideal for the integration of exogenous DNA in *C*. *albicans* [39]. Therefore, they could be useful when it is necessary to resolve the *LAL*, *LHL* or *LUL* markers integrated anywhere in the genome. Since they also induce their own resolution, the resulting stain will only contain a copy of the *loxP* sequence at the *NEUT5L* locus. Although we have not validated every plasmid in this study, all cassettes were sequenced to check the absence of mutations. In summary, the new toolkit generated using the recyclable markers of the *Clox* system represents a significant step forward in the collection of tools available for *C*. *albicans* manipulation that should improve the study and characterization of this major human opportunistic pathogen.

## Materials and methods

### Strains and growth conditions

The strains used in this work are listed in Table A in S1 File. Cells were grown in YPD or in synthetic minimal (SC) medium containing the appropriated supplements at 28ºC. Construction of strains was done using the PCR-mediated procedure previously described [11] using the pFA-derived plasmids generated in this study as template. Transformation of the strains was performed by electroporation [21]. All transformants were checked for correct genome integration by PCR. Correct in-frame insertion of the fluorescent proteins was checked by sequencing or microscopic inspection of the transformants.

### Plasmid assembly strategy

The plasmids in the toolkit were constructed using an assembly strategy that allowed the transfer of the deletion cassettes to the pFA backbone as independent modules. Assembly reactions were performed using the NEBuilder HiFI DNA Assembly Cloning Kit (New England Biolabs) following manufacturer’s instructions. Design of the primers required for PCR amplification of the different modules was performed with the NEBuilder Assembly tool (http://nebuilder.neb.com/). The modules were assembled into the pFA backbone obtained by digestion of plasmid pFA-CaHIS1 with *Bam*HI and *Pme*I [11]. For the deletion plasmids, the selection markers of the *Clox* system (*LAL*, *LHL*, *LUL*, *URA3-Clox* and *NAT1-Clox*) were amplified with primers LXL1 and LXL2 that also carried a common sequence present in the vector, immediately upstream or downstream of the two *loxP* repeats and generated overlapping regions to the pFA backbone to be used in the assembly reaction.

For the assembly of plasmids containing fluorescent proteins or epitope tags, the *Clox* selection markers were amplified with primers GFP-LXL3 and LXL2 that generated overlapping regions between the epitope modules and the 3´ end of the pFA backbone. The different modules containing fluorescent proteins or epitope tags were amplified with a pair of primers that also included overlapping regions with the 5´ end of the pFA backbone and the selection module. For pFA-GFPγ-Clox plasmids, the GFPγ sequence, along with the *ScURA3* terminator was amplified using primers GFP-LXL1 and GFP-LXL2 and plasmid pFA-GFPγ-URA3 [36] as template. The 3xGFPγ sequence with the *ScURA3* terminator present in pFA-3xGFPγ-Clox plasmids and the yEmCherry-*ScURA3* terminator module were amplified using the same primers (GFP-LXL1 and GFP-LXL2) and plasmids pFA-3xGFPγ-URA3 [37] or pFA-yEmCherry-CaURA3 [14] as templates, respectively. To amplify the module containing 3 in-tandem copies of the HA epitope with the S. *cerevisiae URA3* terminator, primers HA1 and GFP-LXL2 were used, with pFA-HA-URA3 as template [15]. For pFA-myc-Clox plasmids, a PCR fragment containing 5 copies of the myc epitope and the *S*. *cerevisiae URA3* terminator was amplified from plasmid pFA-myc-URA3 [15] using primers Myc1 and GFP-LXL2. Finally, the TAP-*ScURA3* terminator module was amplified using primers TAP1 and GFP-LXL2 from plasmid pFA-TAP-URA3 [15]. The modules containing the epitopes or fluorescent proteins were mixed with the recyclable markers and the pFA backbone in independent assembly reactions and transformed in *E*. *coli*. Confirmation of the different plasmids was performed by PCR amplification with internal oligonucleotides and/or restriction digestion. Sequencing of the plasmids to verify the correct sequence was performed at the DNA Sequencing Facility-NUCLEUS of the University of Salamanca.

To construct plasmids pDIS-URA3-Clox and pDIS-NAT1-Clox, plasmid pDIS3 [39] was digested with *Not*I and *Pme*I to release the *NAT1* marker and assembled with the *URA3-Clox* or *NAT1-Clox* modules respectively, amplified with oligonucleotides NEUT7 and NEUT8 that generated overlapping regions for the assembly reaction. To integrate these cassettes at the *NEUT5L* locus, the recyclable markers flanked by *NEUT5L* sequences can be amplified using oligonucleotides N5-up and N5-dw (Table 2). Alternatively, the whole cassette can be isolated by digestion with *Sfi*I (Figure A in S1 File).

The sequences and annotations of all plasmids are available in GenBank. Accession numbers are indicated on Table 1. The plasmids are available from the authors.

### Cre-mediated marker resolution

Elimination of the auxotrophic or resistance markers was performed using a variation of the previously described method [13]. In brief, single colonies of *C*. *albicans* transformants were grown overnight in YPD medium without methionine and cysteine (to induce *MET3p-cre* expression). Cells were plated onto YPD plates and replica-printed onto selective media to select colonies that had lost the auxotrophic and/or resistance markers. The genotype of the resolved mutants was confirmed by diagnostic PCR.

### Western blot

Protein extracts were prepared as previously described [50]. In brief, cells from 5- to 10-ml mid-log cultures were harvested and resuspended in 1 ml of 20% trichloroacetic acid (TCA). The supernatant was removed after centrifugation, and the pellet was resuspended in 100 μl of 20% TCA. Cells were broken using a FastPrep FP120 cell disrupter (BIO 101 ThermoSavant) and the lysate was recovered. Lysates were centrifuged at 1000×*g* for 3 min, and the pellet was thoroughly resuspended in 100 μl of 2×Laemmli buffer and 50 μl of 2 M Tris base. After boiling for 5 min, 10–20 μl were loaded in the gels. Proteins were resolved by 10% SDS-PAGE, transferred to Hybond-P (Amersham Bioscience) membranes and probed with anti-HA High Affinity antibody (3F10, Roche). Horseradish peroxidase-conjugated anti-rat antibody (NA935V, GE Healthcare) was used as secondary antibody. Detection of proteins was performed using the Luminata Forte Western HRP substrate (Merck Millipore).

### Microscopy

Fluorescence microscopy was performed with an Olympus IX81 microscope equipped with a spinning-disc confocal system (Roper Scientific). Three to five z-planes were collected with step sizes of 0.4 μm. Average fluorescence intensity was quantified with ImageJ (http://rsb.info.nih.gov/ij/).

## Supporting information

S1 File Restriction maps of the toolkit plasmids and strains used. Maps and nomenclature of modules cloned into the pFA vector background. *LAL*, *LHL*, *LUL*, *URA3-Clox* and *NAT1-Clox* were used as marker genes. The *GFPγ*, *3xGFPγ* and *yEmCherry* served as fluorescent protein-encoding ORFs. HA, myc and TAP tags are also shown. The annealing sites of the oligonucleotides used for cassette amplification are indicated in blue. Only unique restriction sites are show.
(PDF)
