# Supplementary material for: A new toolkit for gene tagging in Candida albicans containing recyclable markers
Source: PLoS One. 2019 Jul 11;14(7):e0219715. doi: 10.1371/journal.pone.0219715 (PMC6622542; doi:10.1371/journal.pone.0219715)
Supplement: S1 File — Maps and nomenclature of modules cloned into the pFA vector background. LAL, LHL, LUL, URA3-Clox and NAT1-Clox were used as marker genes. The GFPγ, 3xGFPγ and yEmCherry served as fluorescent protein-encoding ORFs. HA, myc and TAP tags are also shown. The annealing sites of the oligonucleotides used for cassette amplification are indicated in blue. Only unique restriction sites are show. (PDF) [file pone.0219715.s001.pdf]

Figure A. Restriction maps of the toolkit plasmids. Maps and nomenclature of modules cloned into the pFA vector background. *LAL, LHL, LUL, URA3-Clox* and *NAT1-Clox* were used as marker genes. The *GFPγ, 3xGFPγ* and *yEmCherry* served as fluorescent protein-encoding ORFs. HA, myc and TAP tags are also shown. The annealing sites of the oligonucleotides used for cassette amplification are indicated in blue. Only unique restriction sites are show.

Figure A

pFA-Clox plasmids (Deletion plasmids)

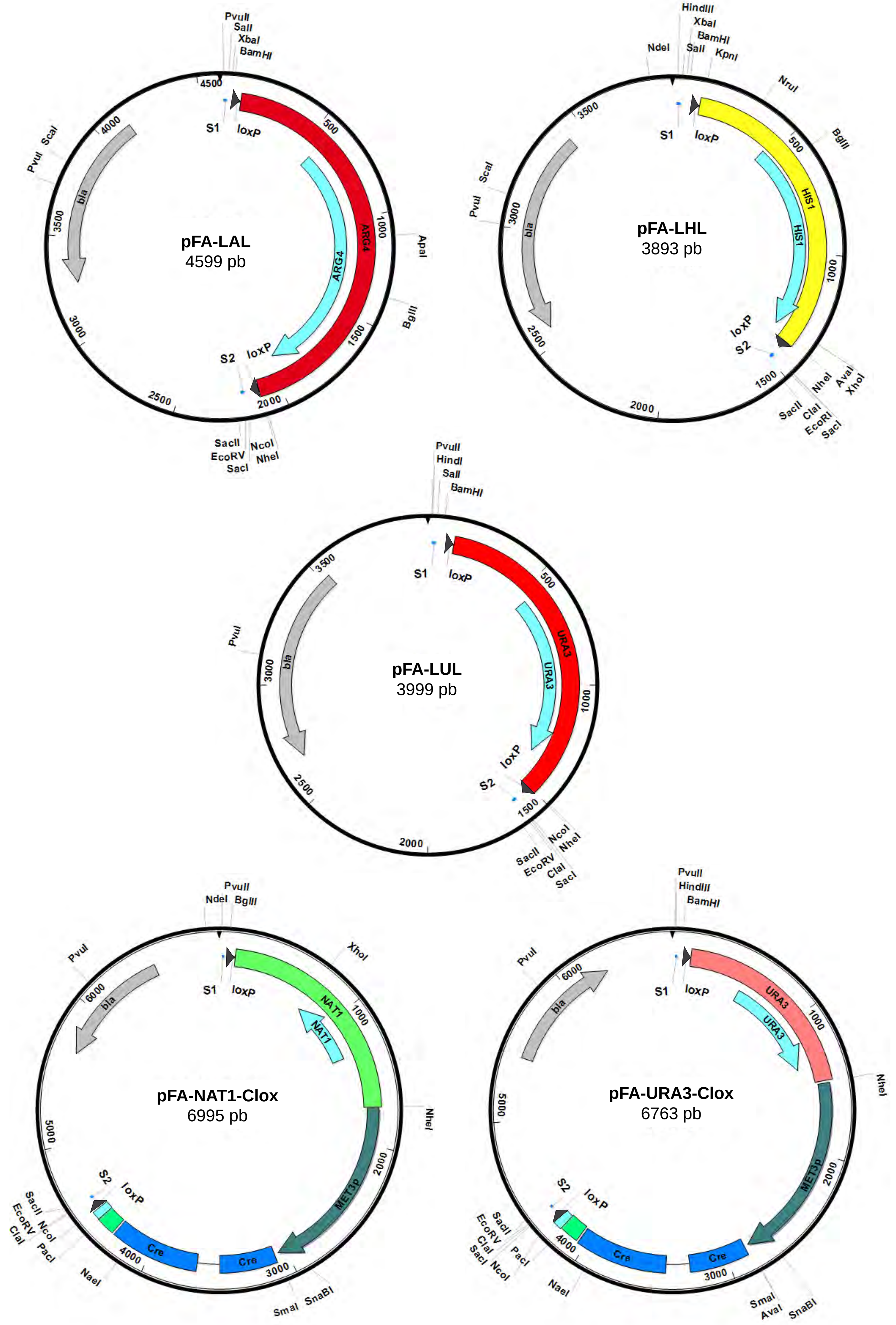

Figure A (continued)
pFA-GFPγ-Clox plasmids

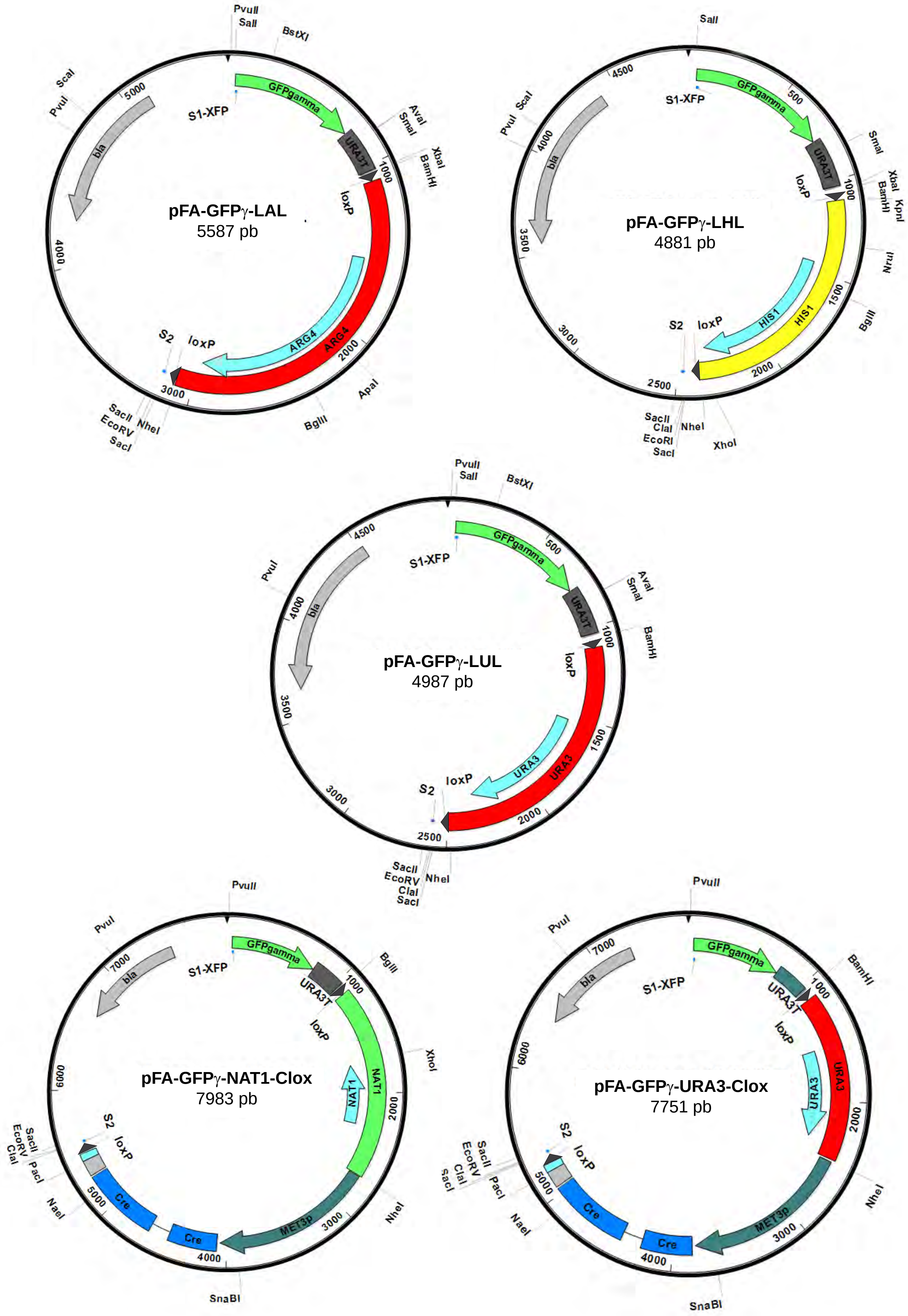

Figure A (continued)

pFA-3xGFPγ-Clox plasmids

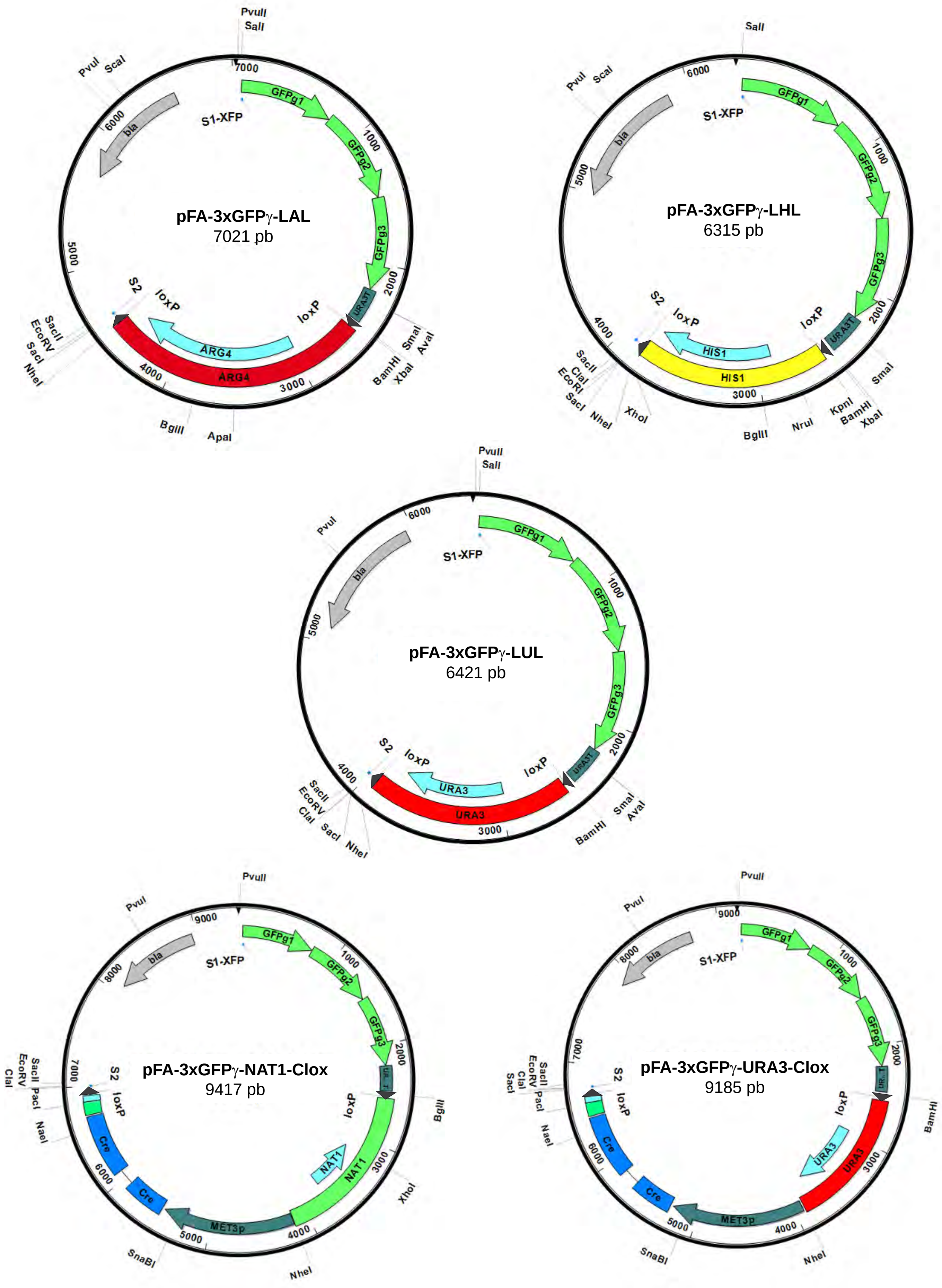

Figure A (continued)

pFA-yEmCherry-XXX plasmids

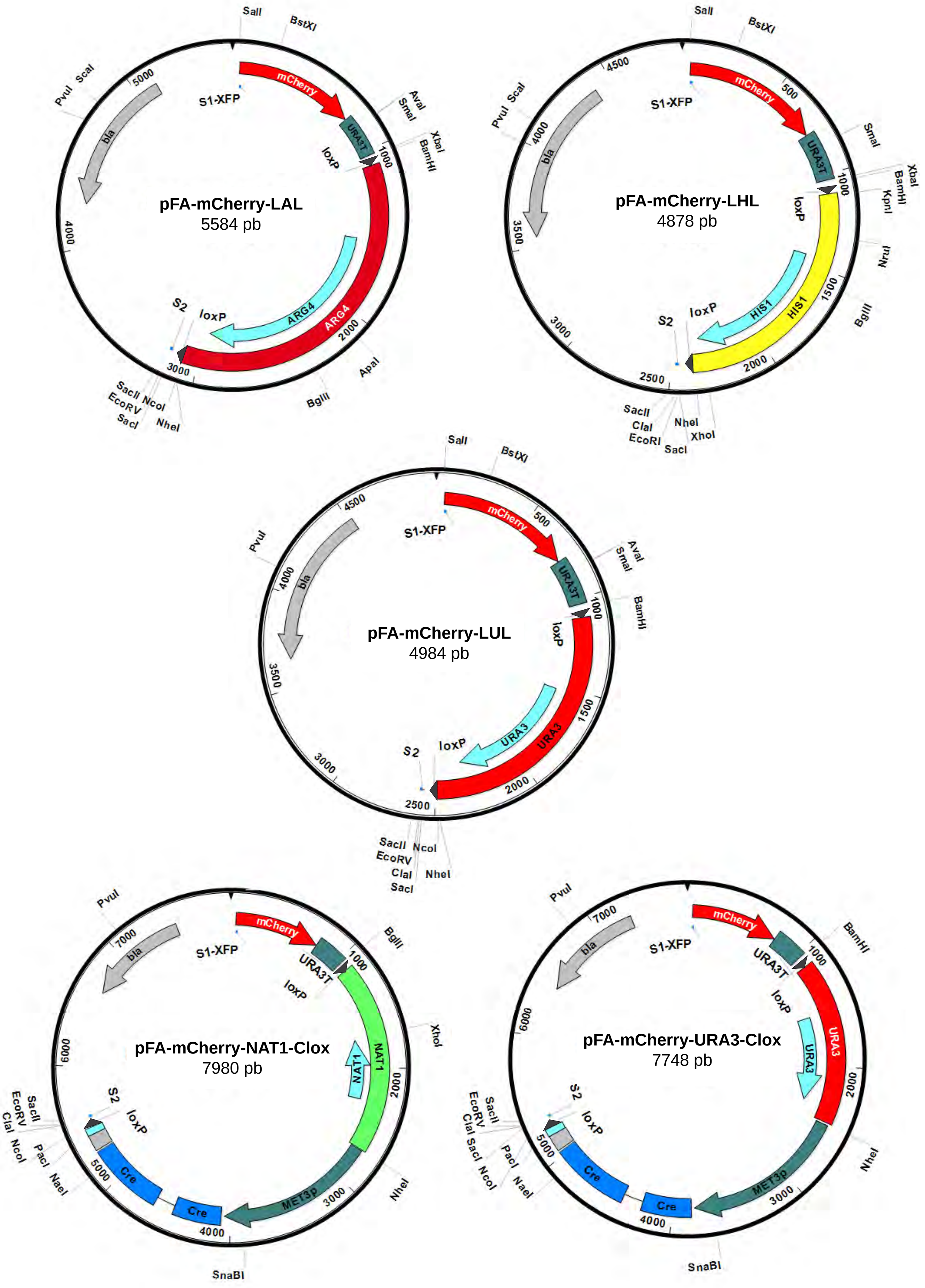

Figure A (continued)

pFA-HA-Clox plasmids

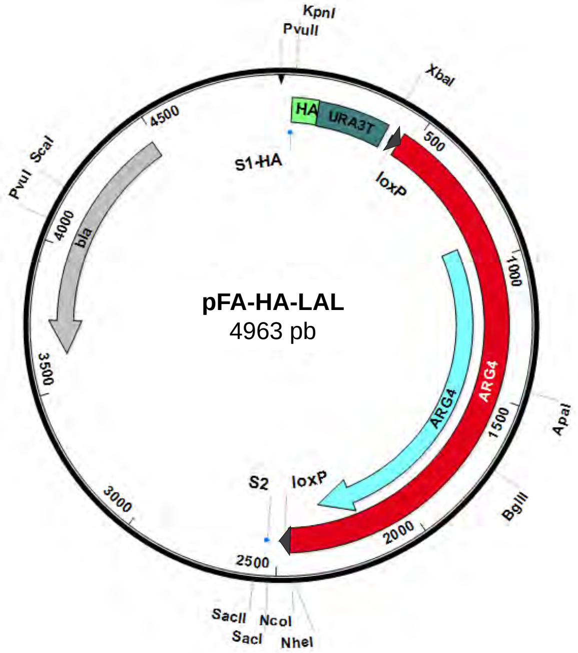

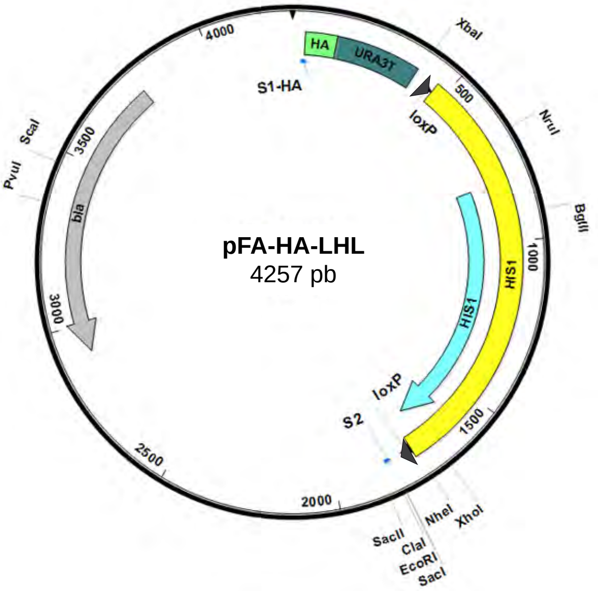

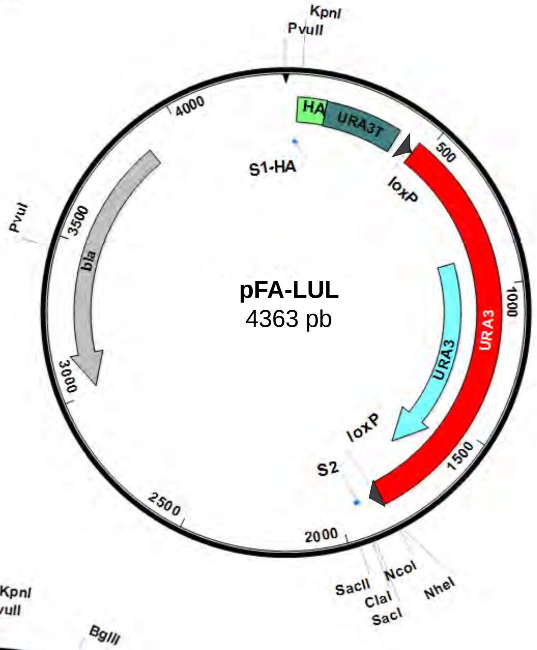

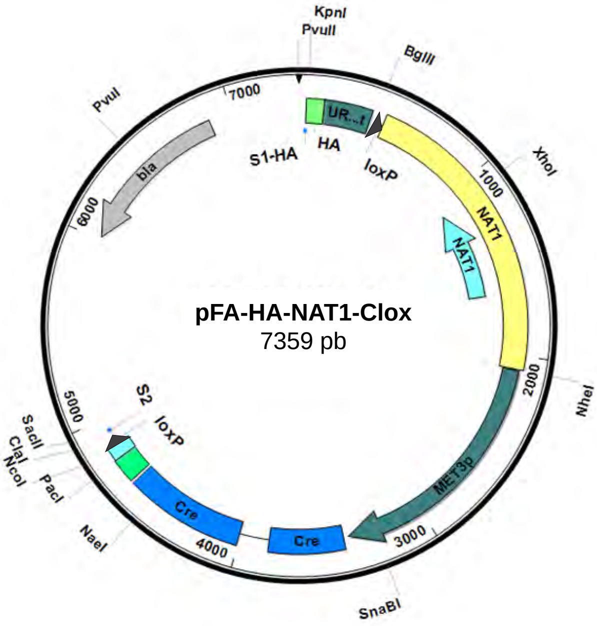

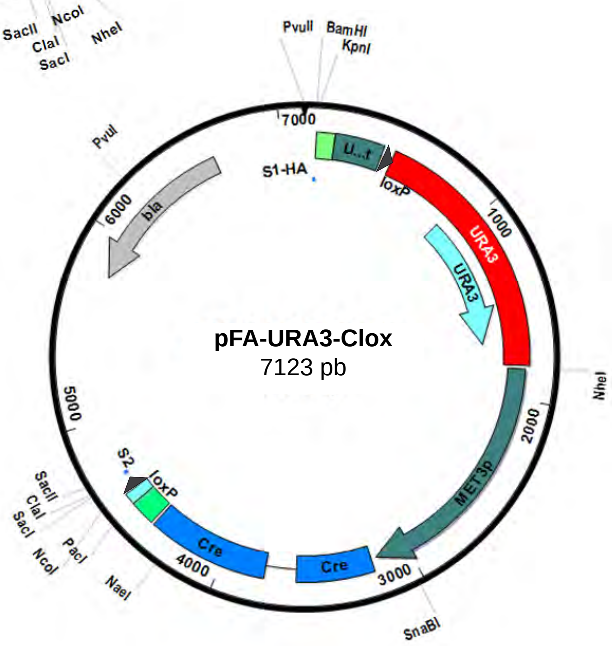

Figure A (continued)

pFA-myc-Clox plasmids

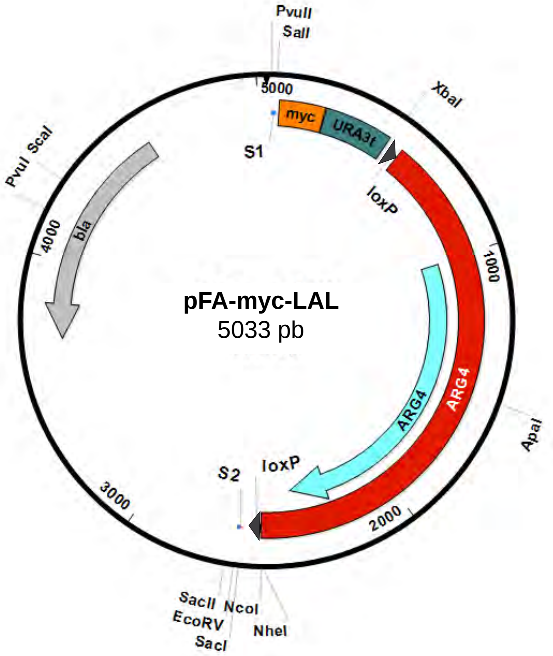

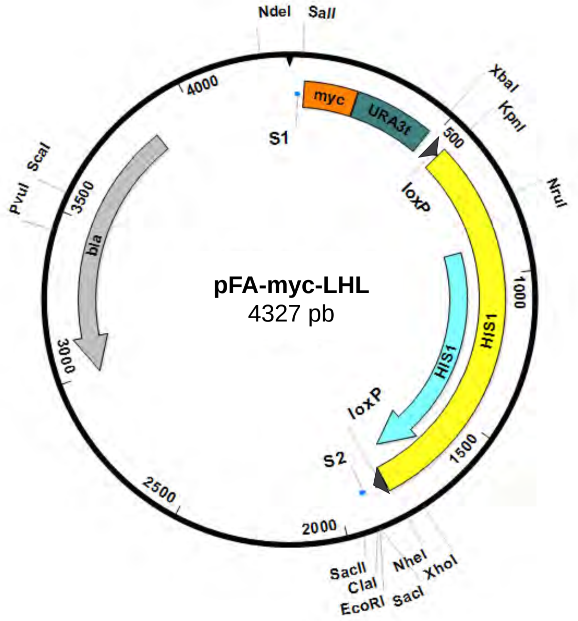

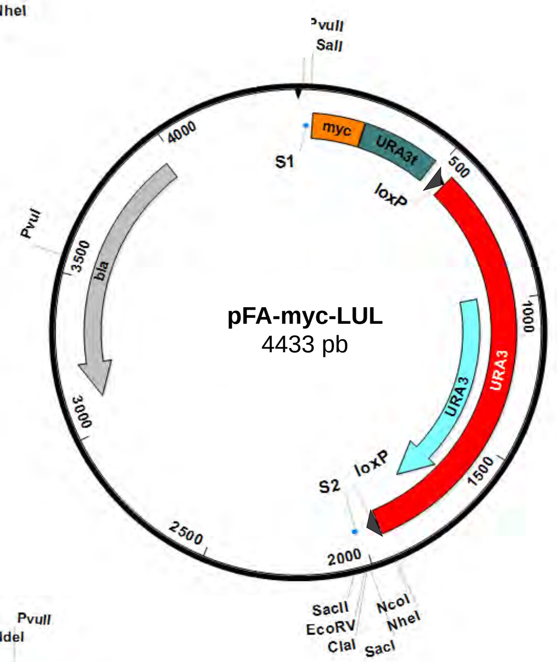

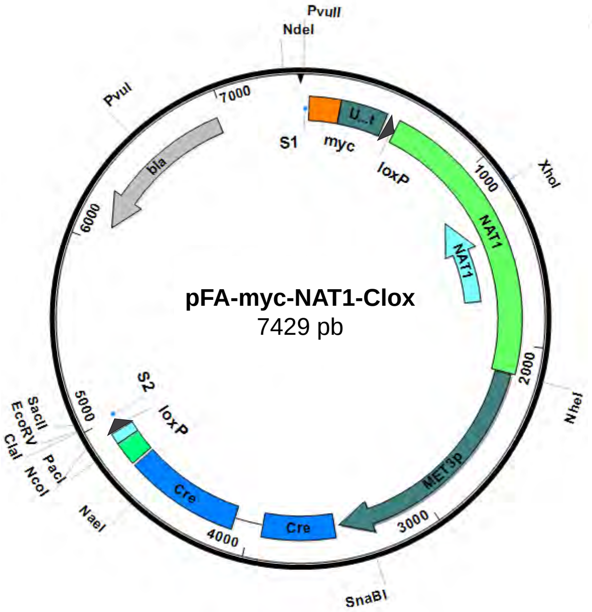

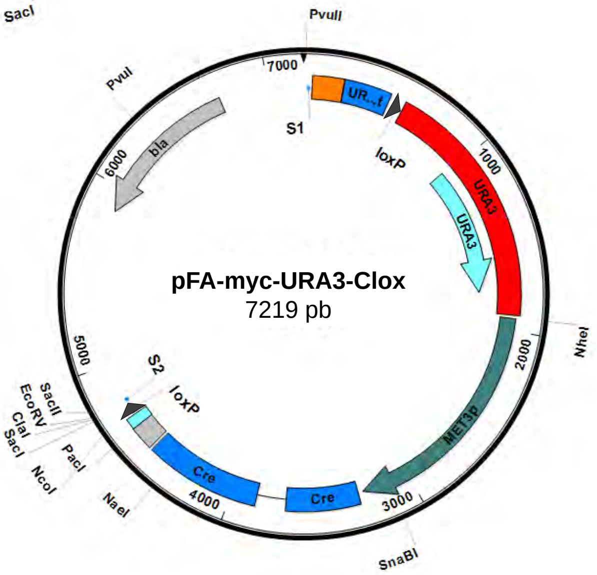

Figure A (continued)

pFA-TAP-Clox plasmids

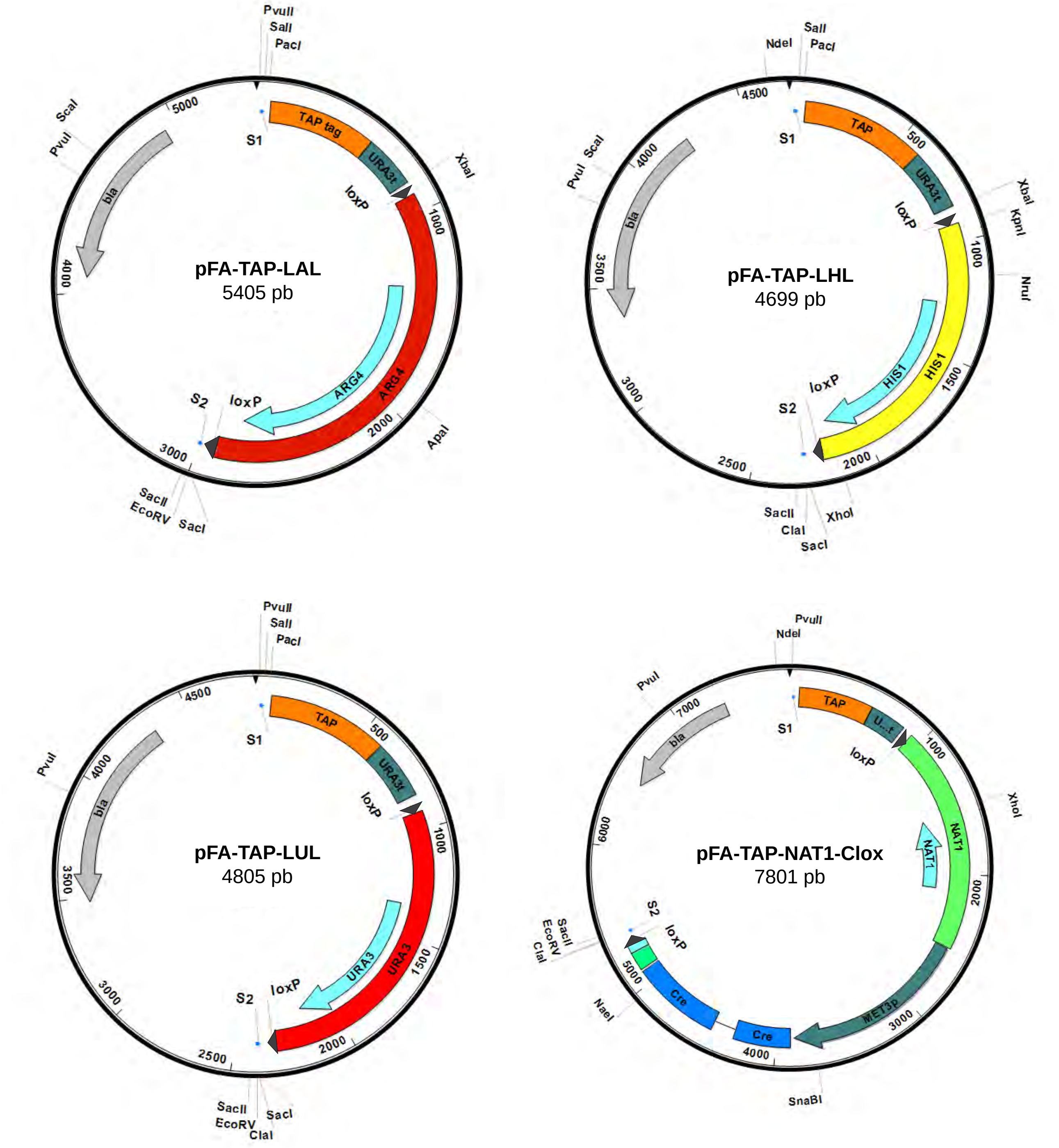

Figure A (continued)

pDIS-Clox plasmids

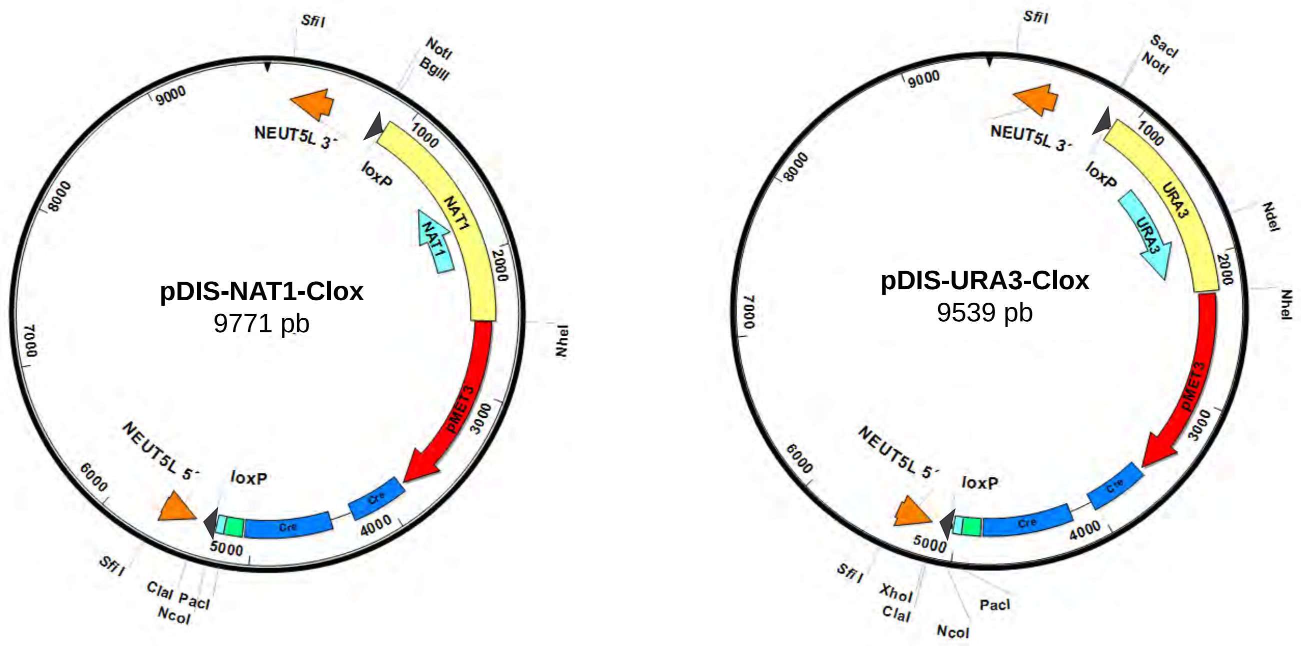

Table A. Strains used

Strain	Genotype	Origin
BWP-17	*ura3Δ::imm434/ura3Δ::imm434 his1Δ::hisG/his1Δ::hisG arg4Δ::hisG/arg4Δ::hisG*	Enloe *et al*., 2000
OL2819	*CDC12-yEmCherry::loxP TUB2-GFPγ::loxP*	This work
OL2835	*SEP7-HA::LAL*	This work
OL2837	*SEP7-HA::LAL CDC10-HA::LHL*	This work
OL2845	*NUP49-GFPγ::LAL/NUP49*	This work
OL2847	*NUP49-GFPγ::LAL/NUP49 TUB2-GFPγ::LUL/TUB2*	This work
OL2866	*NUP49-GFPγ::loxP/NUP49 TUB2-GFPγ::loxP/TUB2 CDC12-GFPγ::loxP/CDC12*	This work
OL2867	*NUP49-GFPγ::LAL/NUP49 TUB2-GFPγ::LUL/TUB2 CDC12-GFPγ::NAT1-Clox/CDC12*	This work
OL2872	*sep7::LUL/SEP7*	This work
OL2908	*sep7::LUL/sep7::NAT1-Clox*	This work
OL2909	*sep7::loxP/sep7::loxP*	This work
CDC12-GFP	*CDC12-GFPγ::LAL/CDC12*	This work
CDC12-3xGFP	*CDC12-3xGFPγ::LAL/CDC12*	This work
TripleHA	*SEP7-HA::LAL/SEP7 CDC10-HA::LHL/CDC10 CDC12-HA::NAT1-Clox/CDC12*	This work
TripleHA resolved	*SEP7-HA::loxP/SEP7 CDC10-HA::loxP/CDC10 CDC12-HA::loxP/CDC12*	This work
